# Developing an ensemble machine learning study: Insights from a multi-center proof-of-concept study

**DOI:** 10.1371/journal.pone.0303217

**Published:** 2024-09-10

**Authors:** Annarita Fanizzi, Federico Fadda, Michele Maddalo, Sara Saponaro, Leda Lorenzon, Leonardo Ubaldi, Nicola Lambri, Alessia Giuliano, Emiliano Loi, Michele Signoriello, Marco Branchini, Gina Belmonte, Marco Giannelli, Pietro Mancosu, Cinzia Talamonti, Mauro Iori, Sabina Tangaro, Michele Avanzo, Raffaella Massafra

**Affiliations:** 1 Laboratorio Biostatistica e Bioinformatica, I.R.C.C.S. Istituto Tumori ‘Giovanni Paolo II’, Bari, Italy; 2 Servizio di Fisica Sanitaria, Azienda Ospedaliero-Universitaria di Parma, Parma, Italy; 3 Fisica Sanitaria, Azienda Usl Toscana Nord Ovest, Lucca, Italy; 4 Fisica Sanitaria, Azienda Sanitaria dell’Alto Adige, Bolzano, Italy; 5 Dip. Scienze Biomediche Sperimentali e Cliniche "Mario Serio", Università degli Studi di Firenze,Viale Morgagni, Firenze; 6 Istituto Nazionale di Fisica Nucleare, Sez. Firenze, Via Sansone 1, Sesto Fiorentino, Firenze; 7 IRCCS Humanitas Research Hospital, Medical Physics Unit of Radiotherapy and Radiosurgery Department, via Manzoni, Rozzano, Milan, Italy; 8 Department of Biomedical Sciences, Humanitas University, via Rita Levi Montalcini, Pieve Emanuele, Milan, Italy; 9 U.O.C. Fisica Sanitaria, Azienda Ospedaliero-Universitaria Pisana, Pisa, Italy; 10 SC Fisica Sanitaria, IRCCS Istituto Romagnolo per lo Studio dei Tumori (IRST) "Dino Amadori", Meldola, Italy; 11 Fisica Sanitaria, Azienda sanitaria universitaria Giuliano Isontina, Trieste, Italy; 12 Fisica Sanitaria, Azienda Socio Sanitaria Territoriale della Valtellina e dell’Alto Lario, Sondrio, Italy; 13 Medical Physics Unit, Azienda USL-IRCCS di Reggio Emilia, Reggio Emilia, Italy; 14 Dipartimento di Fisica Applicata, Università degli Studi di Bari Aldo Moro, Bari, Italy; 15 Centro di Riferimento Oncologico di Aviano (CRO) IRCCS, Via F. Gallini, Aviano, Italy; Islamia University of Bahawalpur: The Islamia University of Bahawalpur Pakistan, PAKISTAN

## Abstract

**Background:**

To address the numerous unmeet clinical needs, in recent years several Machine Learning models applied to medical images and clinical data have been introduced and developed. Even when they achieve encouraging results, they lack evolutionary progression, thus perpetuating their status as autonomous entities. We postulated that different algorithms which have been proposed in the literature to address the same diagnostic task, can be aggregated to enhance classification performance. We suggested a proof of concept to define an ensemble approach useful for integrating different algorithms proposed to solve the same clinical task.

**Methods:**

The proposed approach was developed starting from a public database consisting of radiomic features extracted from CT images relating to 535 patients suffering from lung cancer. Seven algorithms were trained independently by participants in the AI4MP working group on Artificial Intelligence of the Italian Association of Physics in Medicine to discriminate metastatic from non-metastatic patients. The classification scores generated by these algorithms are used to train SVM classifier. The Explainable Artificial Intelligence approach is applied to the final model. The ensemble model was validated following an 80–20 hold-out and leave-one-out scheme on the training set.

**Results:**

Compared to individual algorithms, a more accurate result was achieved. On the independent test the ensemble model achieved an accuracy of 0.78, a F1-score of 0.57 and a log-loss of 0.49. Shapley values representing the contribution of each algorithm to the final classification result of the ensemble model were calculated. This information represents an added value for the end user useful for evaluating the appropriateness of the classification result on a particular case. It also allows us to evaluate on a global level which methodological approaches of the individual algorithms are likely to have the most impact.

**Conclusion:**

Our proposal represents an innovative approach useful for integrating different algorithms that populate the literature and which lays the foundations for future evaluations in broader application scenarios.

## 1. Introduction

In recent years, several kinds of machine learning-based algorithms have been used to address the problems brought on by -omics analyses of clinical data and medical images [1–3]. These models integrate various strategies and methodologies at different stages of their development, from feature selection to data pre-processing until classifier training.

Although encouraging results have been achieved, they have limited application in clinical practice. Frequently in the literature, the models proposed lack evolutionary progression, thus perpetuating their status as self-contained entities. A way to overcome this kind of limitation, is with the development of ensemble predictive models [4–11], a class of techniques that aggregate multiple basic models, thus outperforming the individual constituents.

However, to the best of our knowledge, the ensemble approaches proposed in the literature concern the integration of single algorithms or classifiers and not complex models ranging from data pre-processing to final classification.

In this scenario, we postulated that different algorithms which have been proposed in the literature to address the same diagnostic task, can be aggregated to enhance classification performance.

In this paper, we present the result of a multicenter experience in which each participant independently developed a classification algorithm using the techniques deemed most appropriate by each research group. The trained models were then used to develop an ensemble model to solve a specific diagnostic task. Moreover, we exploited the potentialities of the emerging Explainable Artificial Intelligence (XAI), and we applied a known algorithm to extract additional information for the final user allowing a more reliable use of the instrument [12–16].

Explainable artificial intelligence (XAI) could be closed the gap between the medical professionals and the artificial intelligence algorithms. In the oncological fields, several works have been proposed to explain machine learning and deep learning diagnostic and prognostic outcome [17–23]. In this paper, we applied that approach to explain the result of end-to-end ensemble machine learning model trained to solve a specific medical task independently.

Starting from a public dataset of radiomic features extracted from CT images, each research group implemented its own Machine Learning classification algorithm to predict the lung metastatic state [24].

To further improve the predicted power of the individual models, they were aggregated together via suitable Ensemble Methods like the Classifier algorithm. Afterwards, an algorithm of XAI has been implemented to add new information to the result of the classification generated from the ensemble algorithm to explain which algorithms impacted more on the final result. The innovative approach proposed in this work allows the integration of different algorithms aimed at solving the same search task on the same set of training data. The resulting ensemble model in association with emerging XAI techniques has the potential to define a more robust prediction model on the one hand, and on the other the identification of the individual algorithms that contribute most to achieving the final result. Indeed, XAI algorithm could be useful both to evaluate a result with a borderline classification score more consciously and to evaluate the characteristics of the algorithms that have contributed most to the achievement of the output, generated by the ensemble classifier.

## 2. Materials and methods

### 2.1 Experimental dataset

A public dataset from M. Kirienko et al., of patients who underwent FDG PET/CT in the period 1 January 2011 and 27 June 2017 for the detection of lung nodule [24], was used in the present study. The database includes radiomic features of both CT and PET images of patients with lung lesions, but we chose to focus on the CT ones for this study. The CT cohort consists of 535 patients with lung cancer. Following the histological data, the lung cancers can be divided in metastatic (131 cases, 24.49%) and primary (404 cases, 75.51%). All the clinical data are listed in the Table 1 below.

**Table 1:** Table of the clinical features of the CT dataset. For each feature the absolute value and its frequency is shown.

Clinical Feature	Distribution
*Age (years)*	
(Median; 1^st^-3th quantile)	(67.3; 69.6; 26–90)
*Gender*	
Male (abs; %) Female (abs; %)	(356; 66.7%) (178; 33.3%)
*Scanner*	
Biograph (Siemens) (abs; %)	(234; 43.8%)
Discovery 690 (GE) (abs; %)	(300; 56.2%)
*Histological type*	
*Primary lung tumor (abs; %)*	(404; 75.5%)
*Metastatic (abs; %)*	(131; 24.5%)
*Histological type of primary lung cancer*	
Adenocarcinoma (abs; %)	(190; 35.6%)
Squamous cell carcinoma (abs; %)	(134; 25.1%)
Other primary lung cancer (abs; %)	(80; 19.8%)
*Metastasis site (abs; %)*	
Colorectal (abs; %)	(65; 49.6%)
Lymphoma (abs; %)	(20; 15.3%)
Breast (abs; %)	(14; 10.7%)
Other (abs; %)	(32; 22.9%)

The choice of this specific dataset was dictated by the consistency of the cases and the number of features. Furthermore, the dataset was numerically unbalanced in the two classes of interest (metastatic/non-metastatic). A particular factor that allowed us to make appropriate considerations during the study.

**Fig 1:**
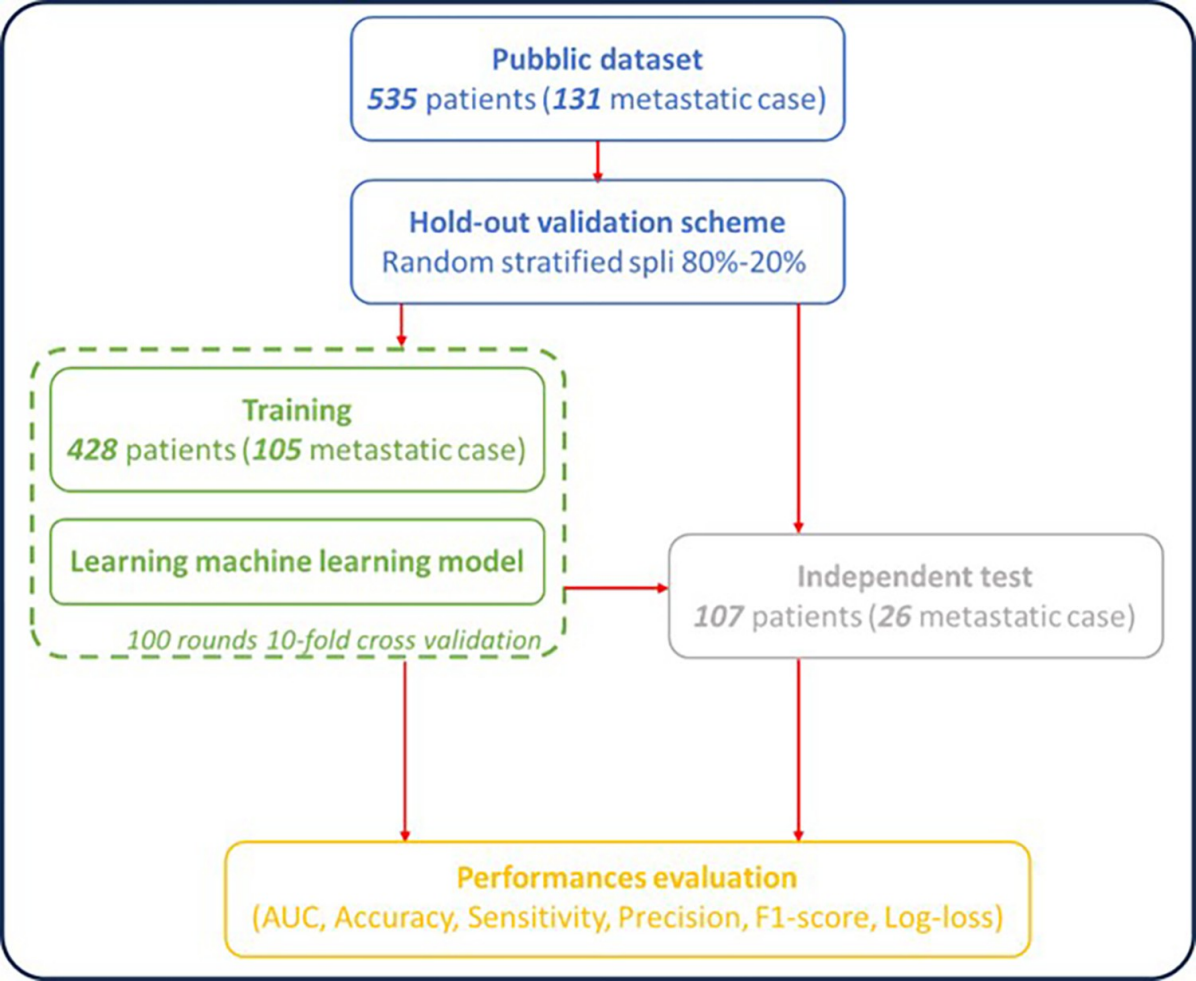
Workflow for implementing machine learning algorithms in the challenge. A same 80:20 hod-out validation scheme was used for all initially trained machine learning algorithms. Additionally, each machine learning algorithm was trained on the training sample and validated in 100 10-fold cross-validation rounds. The algorithms thus defined were validated on the independent dataset. Performances were evaluated for both training validation and independent test, in terms of the Area Under the Curve (AUC), Accuracy, Sensitivity, Specificity, Precision and F1 score.

Seven different algorithms were developed independently by the research groups related to centres involved in this study to differentiate between primary and metastatic lung lesions from CT-based radiomic features. Fig 1 shows the common workflow followed by all the centres, consisting of a split of the starting database in two sub-datasets: one used for training validation and a second one used as independent final test. In our specific case: the entire dataset has been initially split in stratified way between a validation subset of 428 patients (105 metastatic cases), 80% of the total, and a subset for independent test of 107 patients (26 metastatic cases), 20% of the total.

In each model, the validation sub-subset of 428 patients was used for training via a 10-fold cross-validation in 100 external rounds; the small dataset of 107 patients is, instead, used as independent final test.

Performances were evaluated for both training validation and independent test, in terms of the Area Under the Curve (AUC), Accuracy, Sensitivity, Specificity, Precision and F1 score. Moreover, to evaluate how well the classifier ensemble model forecasts the likelihood of each observation as the actual value with respect to the individual algorithms we also calculated the log-loss metric. Log Loss is similar to accuracy, but it will favor models that distinguish more strongly among classes.

Each model was built independently by the developers following the same workflow scheme (see Fig 1). Users were free of using different softwares, platforms, libraries and steps in the machine learning pipeline including the balancing methods, feature selection mechanisms, and classifiers. The implementation code to develop Ensemble model was re-adapted from the Python code at the following link: https://keras.io/exples/vision/and then run by using ColabPro Notebook [25]. A brief discussion of the technical details of the specific algorithm is presented in the Supplementary Material.

### 2.2 Ensemble methods

In the machine learning field, ensemble learning refers to a series of methods that use multiple models or algorithms to achieve better predictive performance than that obtained from the same models applied individually [26, 27]. The approaches known to the state of the art are divided into three fundamental typologies [28]: (a) Bagging: this technique aims to create a set of classifiers each of which will provide a classification result; the final output will be the class that has received the highest number of votes; (b) Boosting: unlike bagging, each classifier influences the final vote with a different weight; the final result will be weighted with respect to the classification accuracy of the individual classifier in the learning phase; (c) Stacking: a further classifier is introduced (called a meta-classifier) which uses the predictions of other sub-models to carry out further learning. Bagging and boosting methods are used to combine algorithms of the same kind, whereas stacking can be used to aggregate algorithms of different types. Therefore, in this paper we used a stacking approach. The classification score generated by each previously independently trained machine learning algorithm became a “feature” for this classifier. This methodology is also called Meta-learning because it is a process of learning from learners in which a higher-level classifier is trained to improve the predictions [29]. The kay principle of meta-learning is that learning proceeds faster with more experience, via the acquisition of inductive biases or knowledge that allows for more efficient learning in the future [30].

**Fig 2:**
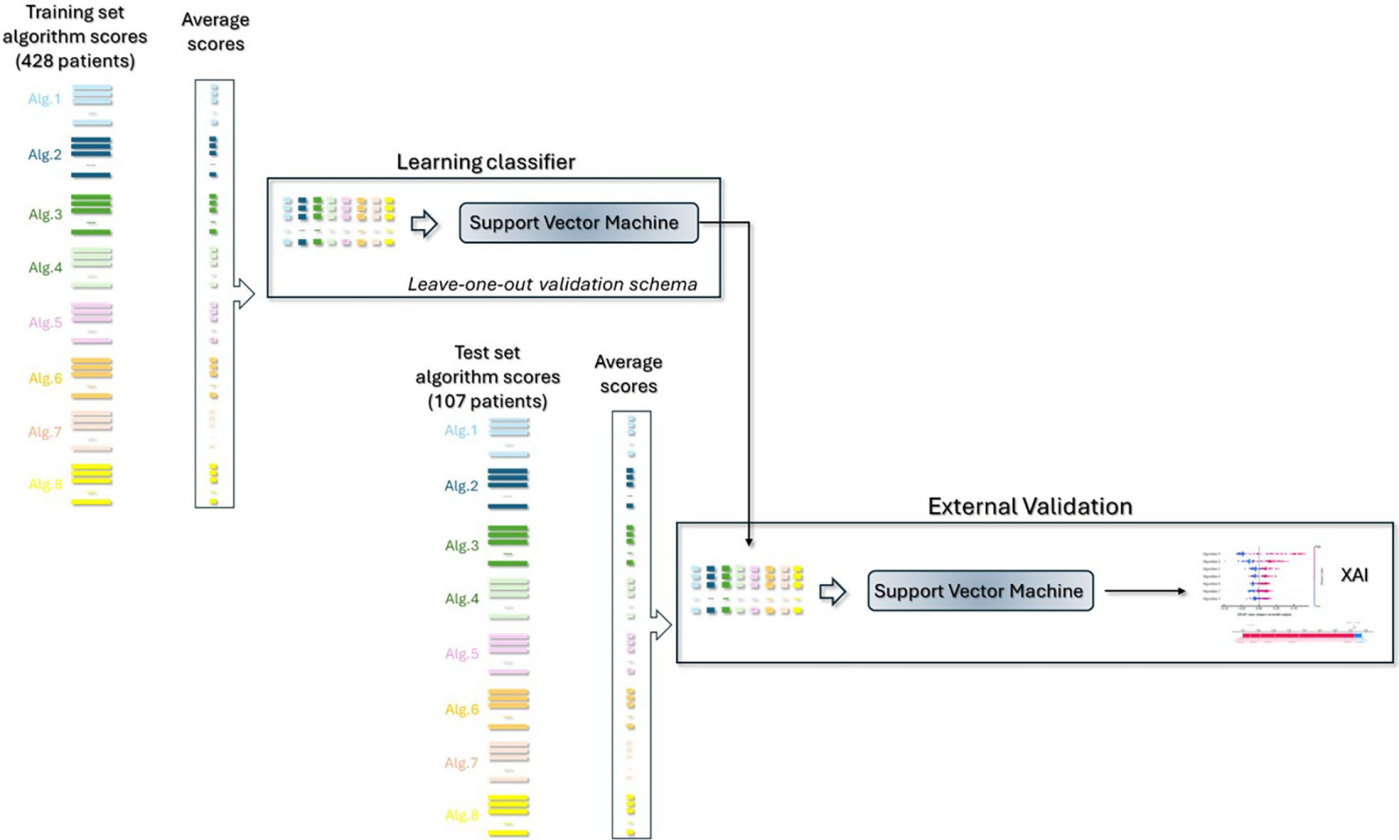
Workflow of the classifier Ensemble method. The scores of the various algorithms were averaged and aggregated; they became “features” of a ensemble machine learning model. Final performances for train and test were evaluated and an XAI approach was implemented to explain which feature-algorithm impacted more on the final predictions.

[29]. As summarized In Fig 2, the general procedure is as follows:

- Each score of the various involved algorithms was averaged and concatenated obtaining final arrays consisting of seven features
- SVM classifier with rbf kernel was used to calculate the training score turned binary via Youden test index, and the final training performances are evaluated [31]. Leave-one-out validation was used to evaluate the model on training set. We also checked that the choice of other classifiers, such as Random Forest, XGBoost, Logistic Regression, did not significantly improve the model;
- The same training validation Youden threshold was then used for the independent test on the subset (107,7) and the final test performances were computed;
- A XAI approach was then adopted to unveil which feature-algorithm impacted more on the final prediction for positive (metastatic patient) and negative cases (no metastatic patient), as described in subsection 2.3.

The other two approaches, such as bagging and boosting, were also evaluated in the experimental phase. However, not having reported better performances also given the preliminary nature of the proposed study, we preferred not to burden the discussion with the exposition of these results.

### 2.3 The explainable algorithm

The XAI mechanism to explain the final classification on a specific patient was implemented via the cutting-edge local explanation SHAP Python package. The SHAP package consists of a local model-agnostic approach using only a classifier’s input and output [32, 33]. In practice, it evaluates the Shapley values, corresponding to the contribution of each algorithm-feature to the final prediction referred to each individual test sample by evaluating each marginal contribution with respect to all the features considered together. For a classification model, the range of the values will be -1 to 1, then because the range of the model output is a probability between 0 to 1. The absolute SHAP value of a feature increases when its weight is larger in defining the classification score.

For the present study we adopted two evaluation tools built on SHAP values. The first tool was the Bee Swarm Plot used to display an information-rich summary of how the features of a dataset impact the model output. It provides a general picture of the SHAP values in the entire reference sample. For each predictor, the distribution of the Shapley values calculated on the reference sample is reported. The colour of each instance is used to display the value of the reference feature.

The second tool was the force plot. For a specific case, the force plot is another way to view the effect each feature has on the prediction. In this plot the positive SHAP values are displayed on the left side and the negative on the right side, as if competing against each other.

## 3. Results

Each algorithm produced validation-training scores matrix with dimension (428,100) and independent test scores matrix (107, 1000) that were then used for the Ensemble method.

**Fig 3:**
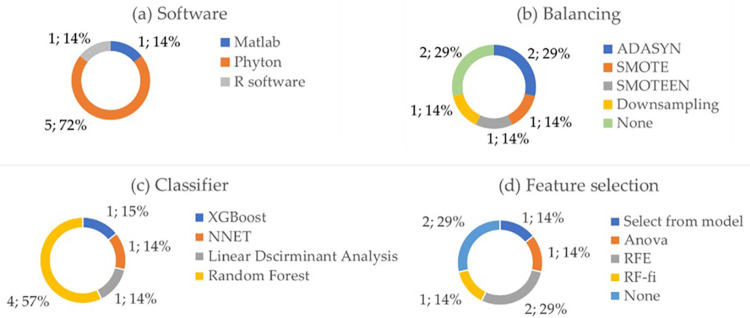
Pie charts of the adopted software (a), balancing technique (b), adopted classifier (c) and feature selection technique (d) by the various algorithms.

Fig 3 shows the pie-charts of the usage of various softwares (a), balancing techniques (b), adopted classifiers (c), and feature selection (d) among the participants in the challenge. Regarding the choice of the software in Fig 3A, 5 algorithms have been developed with Python, 1 algorithm with Matlab and 1 with R software. 5 out of 7 research groups have implemented balancing techniques in their algorithm, such as SMOTE, ADASYN, the hybrid SMOTEEN or downsampling (Fig 3B). In 2 algorithms feature reduction mechanism was not implemented (Fig 5D), and finally, different classifiers were used because they were evaluated as more performing in the analysis phase for the specific algorithm implemented, such as Random Forest, XGBoost, neural network, Linear Discriminant Analysis (Fig 5C).

**Fig 4:**
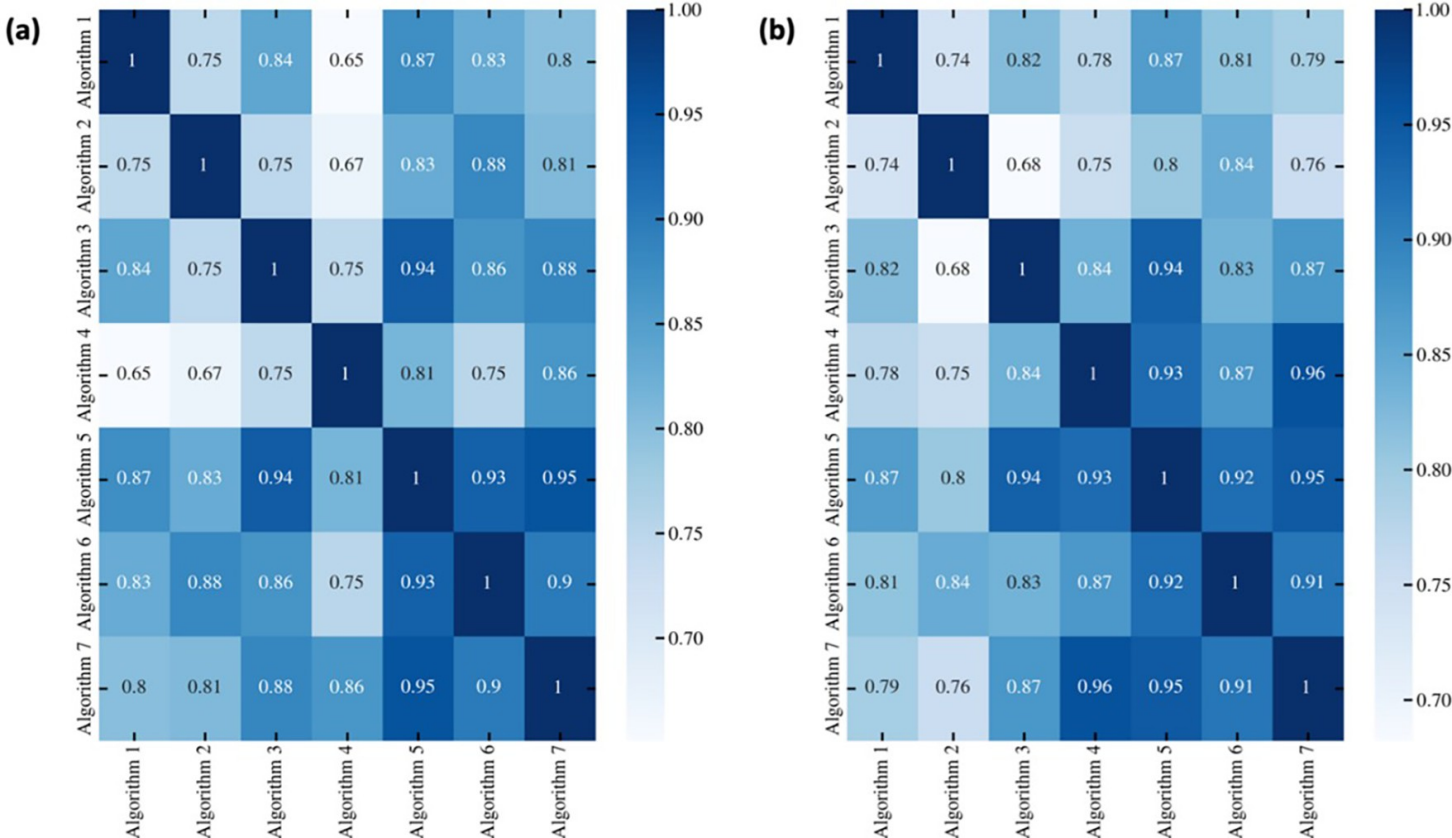
Heatmaps of the correlation coefficients among the classification score of all the seven algorithms for training (a) and test (b).

Fig 4 shows the heatmaps of the correlation coefficient for both training (Fig 4A) and test (Fig 4B). It shows high correlation coefficients among the 7 involved research groups demonstrating that there is agreement between all the algorithms despite the different methods used. All the correlations were statistically significant with p-value < 0.05.

**Fig 5:**
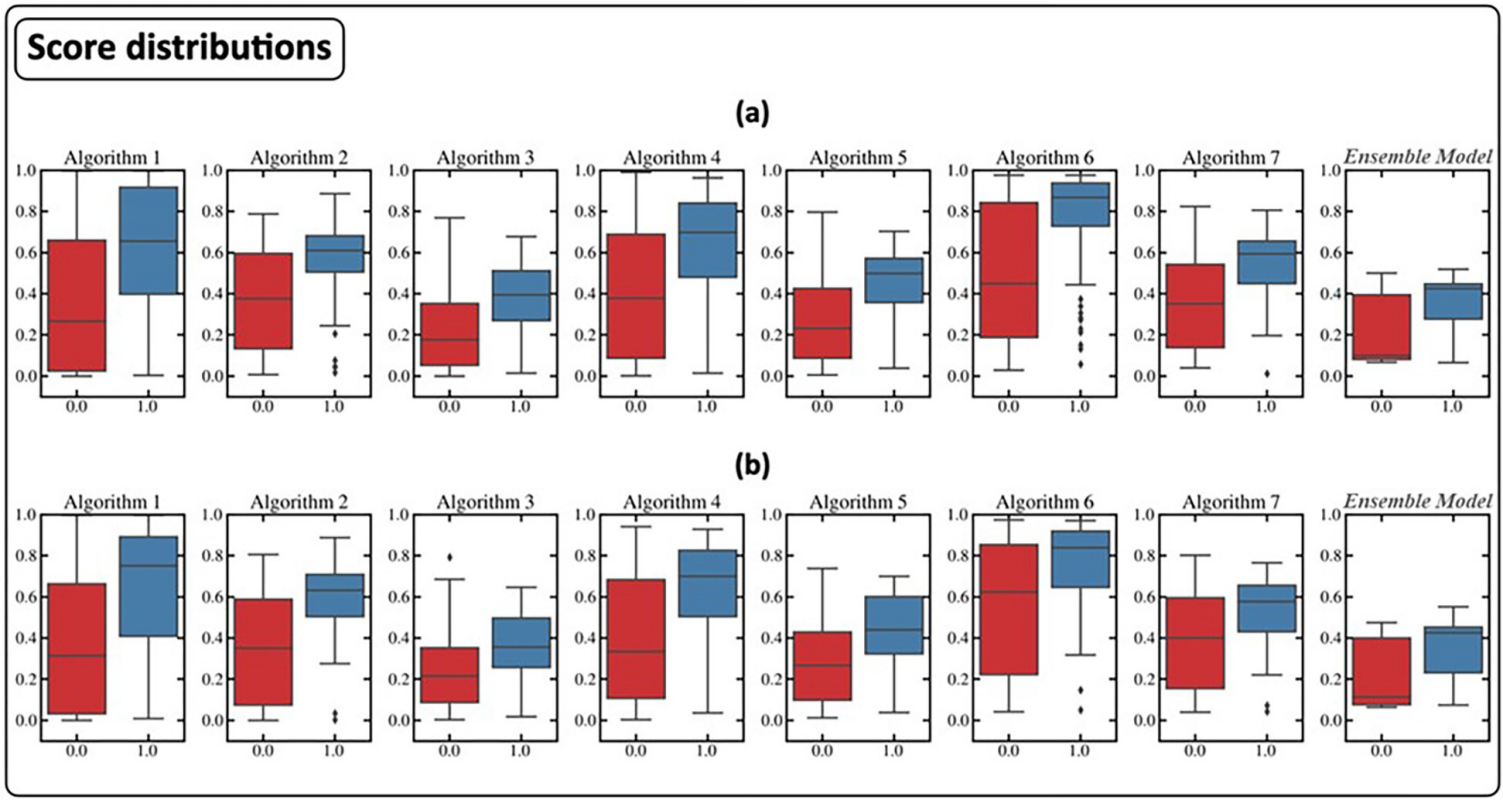
Score distributions for training (a) and test (b) of the various algorithms and the Classifier Ensemble model.

As described in the methods, the average of the scores generated in the different cross-validation rounds of each algorithm became a ’feature’ of the ensemble model. Fig 5 shows the boxplots of the distributions of the scores thus obtained on the training and test set with respect to the label. The distribution of the classification scores generated by the ensemble model is also reported. It should be noted that in our particular case, the median of the score distribution of positive cases generated by the ensemble classifier was significantly lower than that of all the algorithms (p-value <0.05) except for algorithms 3 and 5.

**Fig 6:**
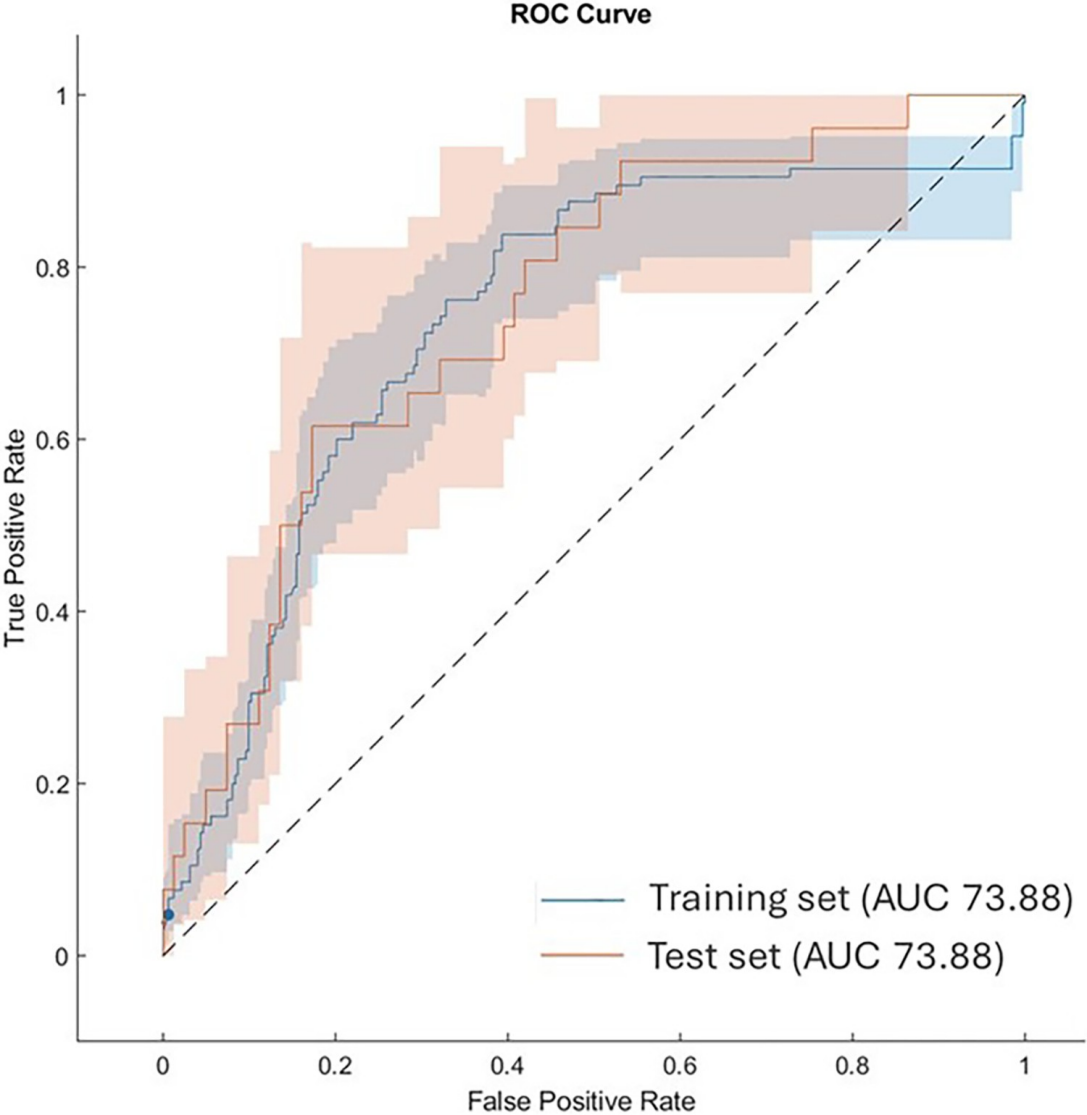
Comparison of ROC curves and the resulting AUC values. Blues curve: Ensemble model in Leave-one-out validation scheme over the training set; Red curve: Ensemble model over the test set. The shaded area around each curve indicates the confidence intervals at 95% level.

Fig 6 shows the comparison of ROC curves and the resulting AUC values on training and test sets. On the training set we found that the optimal threshold was 0.41. The AUC, Sensitivity, Specificity, Accuracy, Precision and F1 score of the Classifier Ensemble SVM Method were 0.74, 0.59, 0.79, 0.74, 0.48, and 0.53 for training, 0.75, 0.62, 0.83, 0.78, 0.53, and 0.57 for the independent test.

**Fig 7:**
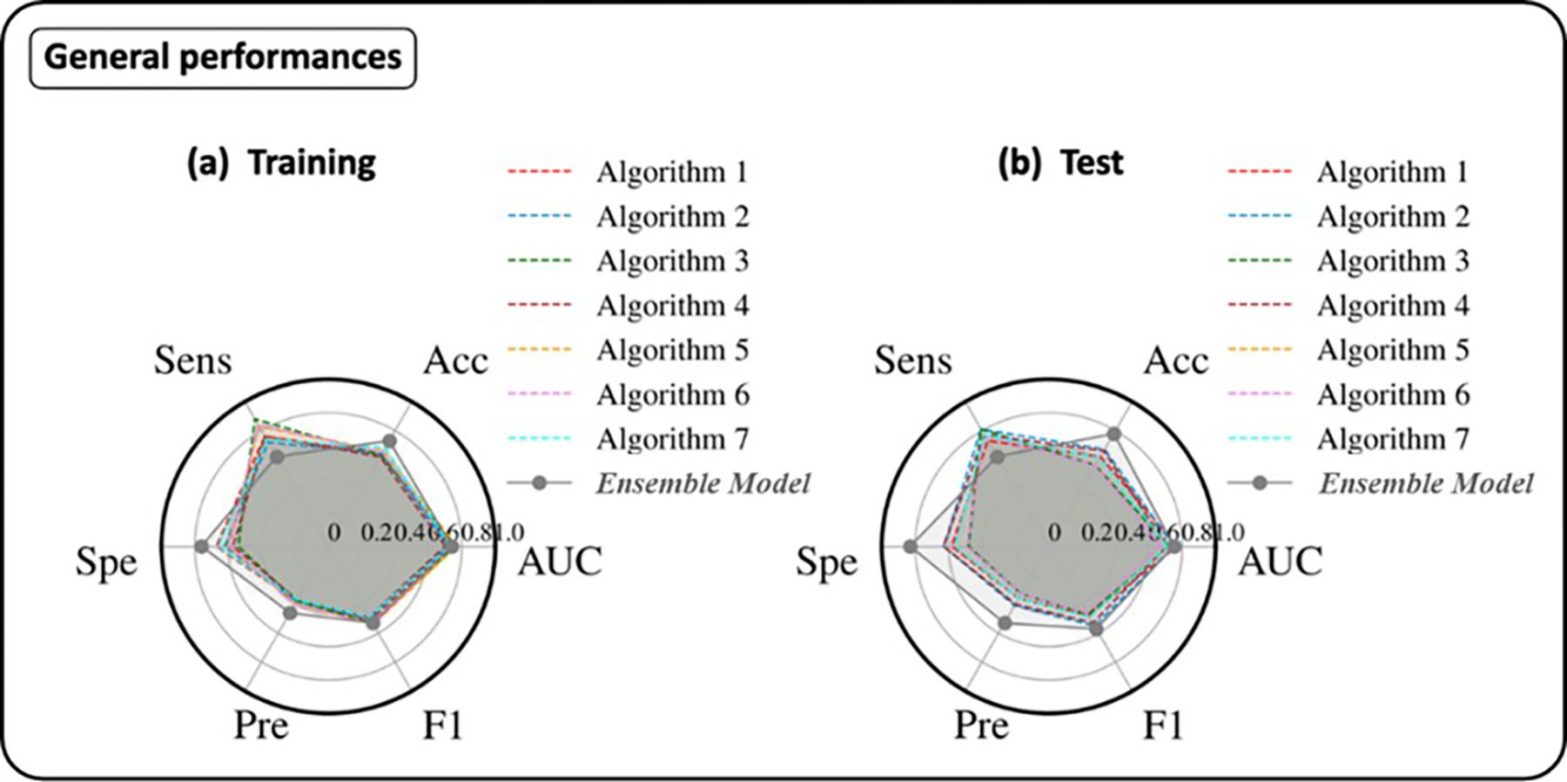
Radar plots of the performances of the various algorithms (dashed lines) and the Classifier Ensemble model for training (a) and test (b). The performance metrics were AUC, Accuracy (ACC), Sensitivity (Sens), Specificity (Spe), Precision (Pre) and F1 score.

Fig 7 shows the radar plots of the performances of all the seven algorithms (dashed lines) and the Classifier Ensemble model both for training (a) and test (b). Compared to the single algorithms, the ensemble algorithm showed an inverted behaviours with respect to the sensitivity and specificity metrics. Probably, this is due to the distributions of classification scores generated by the SVM classifier.

**Fig 8:**
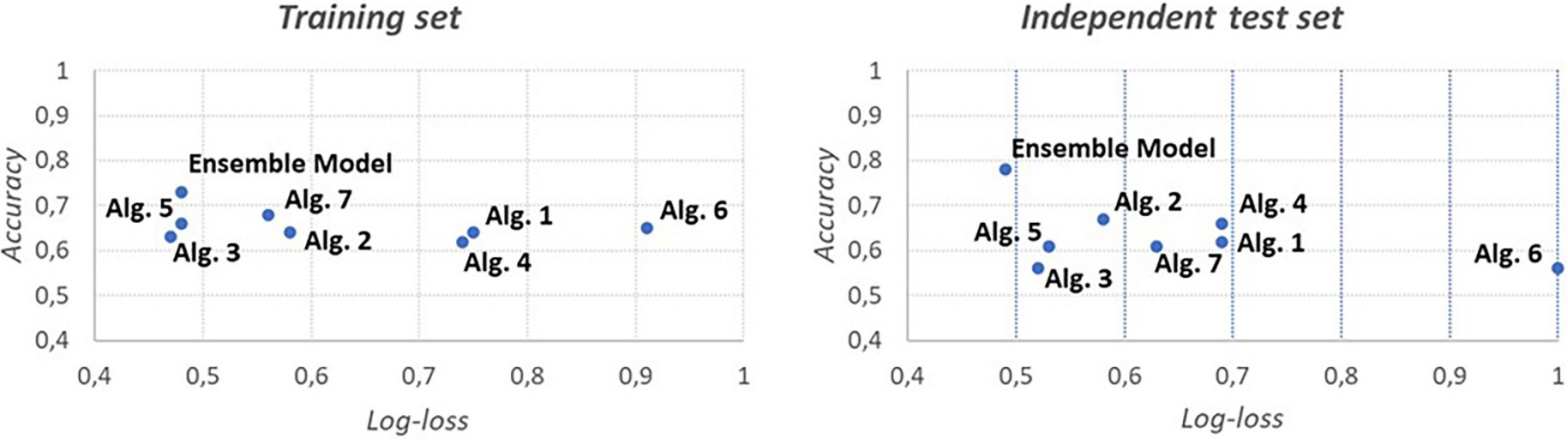
Charts of log-loss metrics for the various algorithms for training and test. Each algorithm has been averaged first and then used for the comparison.

However, accuracy is not indicative of how close the prediction probability is to the corresponding actual/true value. We therefore also calculated the log-loss metric, which is indicative of how close the prediction probability is to the corresponding actual/true value. The log-loss metric shows that the ensemble model outperformed the single algorithms on the test set (Fig 8).

**Fig 9:**
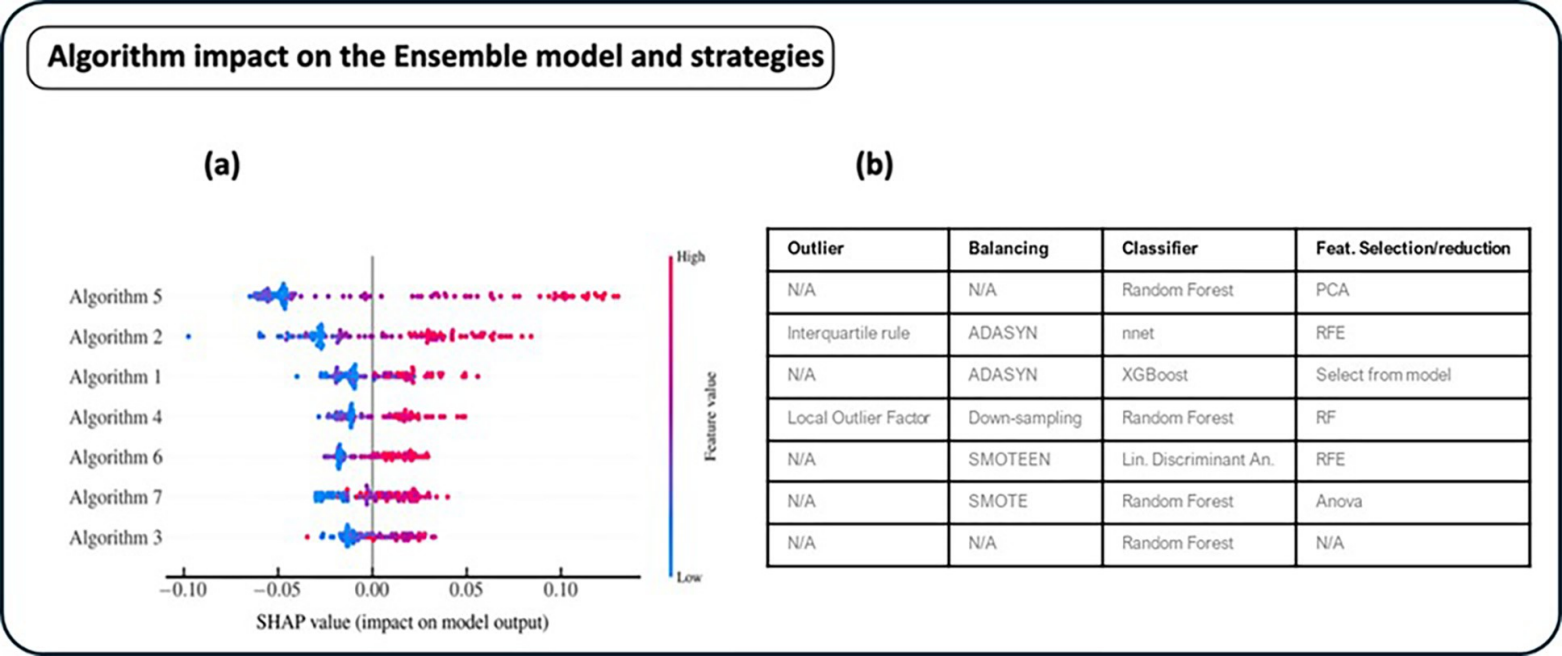
Bee-swarm of the global model (a) and table of the correspondent strategies (b) adopted by the specific algorithm (outlier mechanism, balancing technique, used classifier, and feature selection algorithm).

To identify the algorithms which most impacted on the classification result of the ensemble model, after aggregating the results of all the seven algorithms, we implemented a XAI approach. Fig 9 shows the bee-swarm plot of all the algorithms that contributed more on the positive and negative prediction. Higher (lower) values for each algorithm, indicates a prediction more oriented towards the positive (negative) class. The right bar indicates the value of the feature correspondent to the classification score of the single algorithm. Algorithms 5 and 2 impacted more than the others on the final classification of the Ensemble model. In particular, high classification scores of these two algorithms were more oriented towards the positive (1) class. It should be noted that the strong impact of algorithm 5 justified the lowering of the median classification score of positive cases, which then led to an inversion of the behavior of the sensitivity and specificity classification metrics.

Algorithm 3 is the only one that did not apply any outlier identification, feature selection and dataset balancing techniques. His contribution to the definition of the final score was lower on average.

**Fig 10:**
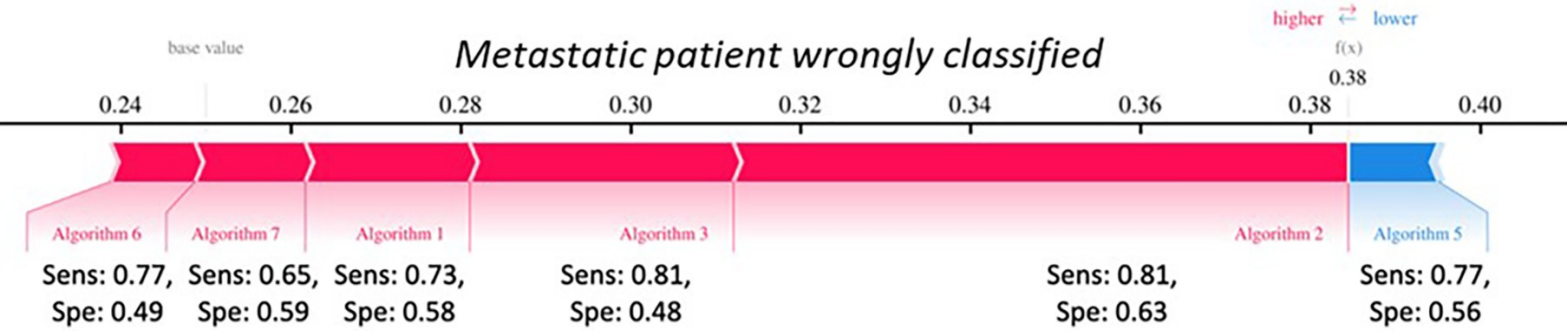
Force-plots of no metastatic sample wrongly classified.

Purely by way of example in Fig 10 is shown the Force-plots of a particular patients. It is a graphical representation of how the individual algorithm-features contributed to achieving the classification score for a specific patient. Specifically, the algorithm features associated with a blue bar reduced (lower) the classification score, i.e. the probability that the patient is metastatic. On the other hand, the algorithm features associated with a red bar increased (higher) the classification score, i.e. the probability that the patient is metastatic. Considering that the cut-off used to binarize the classification result identified by index Youden was 0.40, this case is borderline. This tool would allow for a more accurate assessment of the classification result. Algorithm 5 was the one that pushed the classification score towards the non- metastatic class, however this algorithm had a low specificity (0.58). This tool could provide additional information to the simple classification result, allowing the final user of the model to pay attention to particular cases.

## 4. Discussion and conclusion

Although the last twenty years have witnessed an exponential growth in applications of machine learning models in the oncology field to respond to the main clinical tasks, very few have seen real use in clinical care [34, 35]. Furthermore, the different models proposed to solve the same clinical problem are often heterogeneous and represent neither an evolution of previous models nor an integration of the previous ones. In this paper, we propose an approach that could be used to combine the different relatively accurate models to obtain a better performing and robust model.

The proposed study represented the result of a national challenge promoted by the Artificial Intelligence working group of the Italian Association of Medical Physics. The working group was created with the aim of investigating new methodological ideas for the analysis of biomedical data using artificial intelligence models [36].

Seven research groups have independently developed machine learning algorithms aimed at differentiating between primary and metastatic lung lesions in oncological patients. These algorithms adopted different analysis schemes in terms of data pre-processing, feature selection, classifier. Different data analysis softwarwere used.

We therefore trained a classification model on the scores generated by these algorithms to obtain more accurate and robust performance.

Ensemble models offer several advantages over single models in terms of performance, especially for complex problems and noise [6, 37–39]. By combining multiple trained models to make predictions and using different subsets and predictors of the data, the distribute errors among sub-models of different complexities. Therefore, they can reduce the risk of overfitting and underfitting. In the oncology field, there are various applications of ensemble approaches of machine learning algorithms proposed in the literature [40–44]. However, as far as we know, these proposals only concerned the combination of different machine learning classifiers (SVM, Random Forest, Logistic Regression, etc.) and not of algorithms, which are therefore more heterogeneous under other aspects other than the classifier used, such as the feature selection technique, the balancing method, and the pre-processing operations.

In this paper, we have aggregated the classification scores of the individual algorithms developed by the different research groups using a state-of-the-art classifier. The ensemble model achieved in terms of accuracy, F1-score, and log-loss. On the independent test the ensemble model achieved an accuracy of 0.78, a F1-score of 0.57 and a log-loss of 0.49.

The performance of the ensemble model was significantly better, especially in terms of specificity, also compared to the same work from which the dataset was extrapolated, as reported in Fig 7. The performance of the ensemble model was significantly better, also compared to the same work from which the dataset was extrapolated. In [24], on the validation set, the authors declared an AUC, accuracy, sensitivity, and specificity of 70%, 78%, 95%, and 26%, respectively. Our ensemble model outperformed the proposed model in [24] showing more balanced performances in terms of sensitivity and specificity. Indeed, sensitivity and specificity were equal to 62% and 83%, respectively.

In consideration of the classification results of the proposed individual algorithms and the model proposed in [24], it emerges that the problem of classification from the radiomic features extracted from the same [24] is a hostile one. Although the main objective of the study is not the prediction of metastatic vs. nonmetastatic state, but to define a new approach for the integration of different end-to-end algorithms that is more robust than the individual algorithms, the proposed ensemble model achieves appreciable and superior performance than the individual algorithms.

We have not reported other literature works, because to the best of our knowledge no there are other works on the same reference dataset.Log Loss is useful to compare models not only on their output but on their probabilistic outcome. It should be underlined that in general terms for the evaluation of a classification model the log-loss metric is not an appropriate indicator for evaluating performance on unbalanced datasets. As it is constructed, predicting many low probabilities (associated with negative cases) results in a low log loss value.

Then, we applied the XAI paradigm. The different machine learning applications have already widely shown their potential in different aspects of medicine in the literature. However, especially in the medical field, the lack of transparency of such applications has become increasingly problematic even outside [45, 46]. XAI approach allowed us an added value for the clinical usability of a diagnostic system since it could provide to the clinician a precise evaluation of how the automated system arrived at a given decision [47–49].

For each patient, Shapley values representing the contribution of each algorithm to the final classification result generated by the ensemble model were calculated. This information represented an added value useful for evaluating the correctness/consistency of the classification result on a particular case (patient), for example by looking at the classification performance of the single algorithm which contributed more than others to achieving the final classification result. Furthermore, it also allowed to evaluate on a global level which methodological approaches of the individual algorithms are likely to have the most impact.

This evaluation allows the identification of algorithms with a lower contribution. Reading the distribution of Shapley values associated with the detail of the salient characteristics of the individual algorithms could bring out important considerations. For example, algorithm 3 was the simplest algorithm: no outlier detection, feature selection and balancing techniques had been adopted.

The tools provided by the application of the XAI approach, which were described in a purely illustrative manner in this paper, could have considerable potential in a broader application perspective. The proposed work has limitations related to the nature of the study: what is proposed is a proof of concept conceived in a small working group, but which could be re-proposed by considering different algorithms proposed in the literature. Another limitation is related to the data that have been used: they are retrospective, collected by a single institution at one time point only. Future development will concern the evaluation of the ensemble model generalizability and its comparison with the generalizability of individual algorithms. Nonetheless, we believe that what has been proposed in this paper represents an innovative approach useful for integrating different algorithms that populate the literature and which lays the foundations for future evaluations in broader application scenarios. It is important to clarify that now the integration of multiple algorithms that comes from literature cannot systematically implemented due to the heterogeneity of surrounding conditions not related to data processing and analysis. They could be identified in discrepancy on data acquisition methodologies, patient inclusion criteria, endpoint definition and more. However, the availability of approaches for the integration of multiple machine learning algorithms from literature could spontaneously place a new requirement for a tighter definition of study designs, their sharing and compliance.

## Supporting information

S1 File (DOCX)
