# Supplementary material for: Developing an ensemble machine learning study: Insights from a multi-center proof-of-concept study
Source: PLoS One. 2024 Sep 10;19(9):e0303217. doi: 10.1371/journal.pone.0303217 (PMC11386419; doi:10.1371/journal.pone.0303217)
Supplement: S1 File — (DOCX) [file pone.0303217.s001.docx]

**Supplementary Material:**

In this subsection, the various algorithms of the involved centers are briefly described.

- - 1. Algorithm 1

A machine learning pipeline was implemented in python language which was executed in the Colab cloud-based environment. It included XGBoost classifier with larger weight for positive scores, ADASYN for oversampling, and Robust Scaling for features scaling. Feature selection was performed using SelectFromModel which selects features according to their importance in the XGBoost classifier.
The hyperparameters number of features to be selected, number of neighbours in the ADASYN, and learning rate in the feature selection classifier were chosen using search CV. All the algorithms were from the Scikit-learn libraries [50]. Metrics were evaluated using cross validation on the train set and on the test set. The entire procedure (5-fold CV + train evaluation) was repeated 10 times.

- - 1. Algorithm 2

The algorithm of Center 2 was developed using the packages Caret [51] and Imbalance [52] of R software.

A 10-fold cross validation was repeated 100 rounds on training set data. The analysis pipeline included the following steps: RFs standardization (z-score), redundant RFs detection (cross-correlations between features with R>0.7) and elimination, outlier detection and removal using the interquartile range rule, endpoint balancing by means of the Adasyn algorithm, feature selection via recursive feature elimination (wrapper) and finally the train of the classifier nnet (feed-forward neural networks with a single hidden layer). Model parameters of nnet were the number of units in the hidden layer and the weight decay. They were determined based on training performances and then fixed over all 100 rounds. Their final values were number of units in the hidden layer equal to 60 and weight decay equal to 0.56.

In each rounds at the end of the 10-fold cross validation the model was re-trained on the whole training set and then tested on unseen data that belonged to test set. The performance metrics (AUC, Accuracy, Sensitivity, Specificity, Precision, and F1 score) were evaluated as average values among the 100 rounds with the associated standard deviations. The predefined threshold of 0.5 was used for the calculation of performance metrics.

- - 1. Algorithm 3

The algorithm of Center 3 was built in Matlab R2022b platform, using the ‘Statistics and Machine Learning Toolbox’. The classification algorithm was Random Forest, implemented using the default parameters of ‘fitcensemble-bag’ function and with 200 classification trees. No balancing of data, no tuning of parameters and no feature selection were employed. A 10-fold cross-validation, repeated 100 times, was applied on the training validation subset. Nested in the cross-validation, z-score method was implemented to standardize data. Both the validation and the test subsets were standardized consistently with the training set. The 1000 models obtained in the training validation phase were used to classify the subjects of the independent test subset, hence a matrix of (107,1000) classification scores was obtained.

- - 1. Algorithm 4

The algorithm of Center 4 was built with Python Scikit-learn and Imbalanced Learn libraries [53].

Inside the 10-fold cross validation, the training subset of 428 patients was first preprocessed removing the local outliers, using the Standard Scaler, and balanced via Neighborhood Cleaning Rule under sampling technique [28].

Then, a feature selection mechanism was adopted randomly splitting the training subset fold in 80%-20% portions and building a feature importance vector via Random Forest feature importance.

The features with value greater than the average value of the importance array was considered and only the ones with a frequency greater than a threshold of 0.7 were selected as relevant.

Then a Random Forest classifier with 100 tree and 5 leaves was used. Via Youden test index the optimal threshold to binarize the training cores was performed, and training performances were calculated [28].

The same Youden training threshold was also used for the independent test.

- - 1. Algorithm 5

The algorithm of center 5 was built with Python Scikit-Learn [53] and Optuna [54, 55] packages.

This algorithm is based on a pipeline composed by a features scaling step, a dimensionality reduction step and a Random Forest as a classifier. Through a Bayesian optimization strategy performed by the Python package Optuna we select the best features scaling algorithm (between MinMax, RobustScaler, StandardScaler), the best dimensionality reduction strategy (between PCA, Mutual Information and no dimensionality reduction) and the best hyperpameters of the Random Forest.

The optimization was performed in a 10-fold CV strategy, the optimized pipeline was then trained on the entire training set and performances on the training and test set were computed.

The entire procedure was repeated 10 times.

- - 1. Algorithm 6

The pipeline was implemented with Python 3.10.8, using the machine learning libraries scikit-learn 1.1.0, imbalanced-learn 0.10.1, and xgboost 1.5.0. All the code was executed on the high-performance computing cluster of IRCCS Humanitas Research Hospital.

The pipeline performs the following steps:

1. Robust scaling of feature values f:

2. Feature selection using recursive feature elimination with 5-fold cross-validation. The linear

discriminant analysis algorithm was used to provide information about feature importance

3. Class balancing with synthetic minority oversampling technique and cleaning using edited

nearest neighbors

4. Classification using xgboost algorithm

The best hyperparameters of the pipeline were selected by randomly sampling the search space † 1000 times and performing a 10-fold cross-validation for each iteration. Then, 10 rounds of 10-fold cross-validation were executed using the best hyperparameters found in the previous step. All cross-validations were performed in a stratified manner.

- - 1. Algorithm 7

This algorithm uses random forest as classification algorithm and SMOTE as up sampling strategy. The parameters of the Random Forest were choosen using a randomized search CV implemented in Scikit-learn libraries [50]. For each step of the 5-fold cross validation the metrics were evaluated on the test subset. Reported test metrics were then averaged over all steps. Metrics were evaluated on the train set. The entire procedure (5-fold CV + train evaluation) was repeated 10 times.
